# Microporous poly- and monocrystalline diamond films produced from chemical vapor deposited diamond–germanium composites

**DOI:** 10.1039/d2na00688j

**Published:** 2023-01-24

**Authors:** Vadim Sedov, Artem Martyanov, Alexey Popovich, Sergey Savin, Dmitry Sovyk, Ivan Tiazhelov, Dmitrii Pasternak, Soumen Mandal, Victor Ralchenko

**Affiliations:** a Prokhorov General Physics Institute of the Russian Academy of Sciences Moscow 119991 Russia sedovvadim@yandex.ru; b Kotel'nikov Institute of Radio Engineering and Electronics RAS Fryazino 141120 Russia; c MIREA – Russian Technological University Moscow 119454 Russia; d School of Physics and Astronomy, Cardiff University CF24 3AA Cardiff UK mandals2@cardiff.ac.uk soumen.mandal@gmail.com; e Harbin Institute of Technology Harbin 150001 P. R. China

## Abstract

We report on a novel method for porous diamond fabrication, which is based on the synthesis of diamond–germanium composite films followed by etching of the Ge component. The composites were grown by microwave plasma assisted CVD in CH_4_–H_2_–GeH_4_ mixtures on (100) silicon, and microcrystalline- and single-crystal diamond substrates. The structure and the phase composition of the films before and after etching were analyzed with scanning electron microscopy and Raman spectroscopy. The films revealed a bright emission of GeV color centers due to diamond doping with Ge, as evidenced by photoluminescence spectroscopy. The possible applications of the porous diamond films include thermal management, surfaces with superhydrophobic properties, chromatography, supercapacitors, *etc.*

## Abstract 2

Microwave plasma assisted chemical vapor deposition was used for the formation of diamond–germanium composite films. By selective removal of the Ge phase, a series of porous polycrystalline and single-crystal diamond films and membranes were obtained.

## Graphical abstract

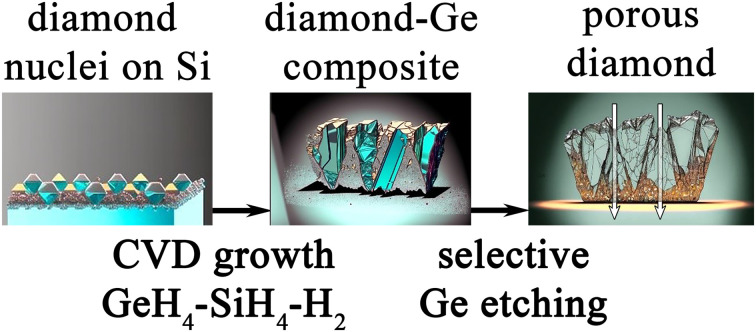

## Introduction

Due to its exceptional electronic, optical, thermal and mechanical properties, diamond is the material of choice for important applications in active and passive electronics, lasers, quantum photonics, radiation detectors, superhard coatings on tools, and many others.^1–8^ A special sort of diamond, porous diamond films and crystals, is of increasing interest for use in certain applications where a high surface-area-to-volume ratio is necessary. Such a porous diamond has a wide range of potential applications in electrochemistry,^9–13^ supercapacitors,^14–18^ robust filters,^19–21^ 3D detectors of ionizing radiation,^22^ an efficient adsorbent for hydrogen storage^23^ and solid-phase extraction and high-performance liquid chromatography.^24^ The porosity of the diamond surface also can be used to tune the wettability of diamond materials.^25–27^

The porosity of diamond crystals, films, membranes and compacts can be achieved by a variety of methods. The most obvious top-down approach is based on post-growth treatment of solid micro-nanocrystalline diamond films such as laser drilling,^25,28^ reactive ion etching,^29–31^ gas-mediated electron beam-induced etching,^32^ and chemical etching of grain boundaries in nanocrystalline diamond films.^33–35^ A local etching of diamonds with catalytically active metals like Fe and Ni to pattern diamond films and single crystals, particularly for hole formation, was used in a number of studies,^36–42^ in which case, the treatment takes place in a hydrogen atmosphere at a temperature of about 900 °C. The metal particles placed on the diamond surface dissolve carbon, thus penetrating the interior of the material. The advantage of the top-down approach is the possibility to choose an initial diamond material, including a single-crystal diamond. However, all etching-based techniques face difficulties if a high aspect ratio of small-sized pores is needed (*e.g.* see ref. 40).

Another simple method to obtain a porous diamond, usually nanocrystalline or microcrystalline, is to produce diamond-based ceramics or composites followed by chemical etching of the non-diamond phase. Bulk porous polycrystalline diamonds have been produced by a compaction process of undoped and boron-doped microcrystalline diamond particles under high pressure-high temperature (HPHT) conditions.^13^ Ekimov *et al.*^43^ reported HPHT synthesis of diamond–copper composites, that were converted to a porous diamond skeleton by selective etching of the Cu binder. In a recent work by Wang *et al.*, millimetre-scale single-crystal diamond particles with porous diamond layers were obtained with a modified HPHT technique.^44^ However, the HPHT diamonds are limited in size and cannot be prepared in the form of thin films, so another technique for the synthesis of various diamond composites can be used instead – chemical vapor deposition (CVD).^45–47^

CVD growth of diamond usually results in continuous polycrystalline or single-crystal films with no gaps or pores. However, CVD can also be used for direct deposition of diamond on porous templates, such as Cu meshes,^26^ Cu or Ni foams,^48,49^ porous Ti substrates,^50^ SiO_2_ fibers on top of planar B-doped diamond films,^51^ porous polypyrrole scaffolds,^52^ “forests” of vertically aligned multiwalled carbon nanotubes,^53^ buckypapers composed of single-wall carbon nanotubes,^54^ porous Si,^55^ irregularly arranged SiO_2_ microspheres,^17^*etc.* Moreover, the use of highly ordered templates allows the fabrication of structured diamond films, such as inverted diamond opals.^56,57^ But, the template-based approaches have a common downside, the part nearest to the plasma has a higher growth rate then the parts further away. As a result, a fully coalesced film is quickly formed in these parts denying plasma access to pores deep inside the template. This effect puts a limitation on the possible thickness of the porous diamond layer which can be grown using the template technique.

In order to solve this problem, Zhuang *et al.*^16^ were first to report on a “template-free” approach for fabrication of three-dimensional diamond networks, which combines the deposition of a diamond/β-SiC nanocomposite film with a wet-chemical selective etching of the β-SiC phase. This approach is based on the deposition of diamond/SiC composite films by CVD using H_2_–CH_4_ mixtures with added tetramethylsilane (TMS) as the silicon precursor.^58^ However, the growth rate of such composites is rather low (100–300 nm h^−1^),^59–61^ which complicates the synthesis of thick (>10 μm) porous films. In addition, the chemical properties of β-SiC are quite close to those of a diamond, and thus the selective removal of the SiC phase with wet chemistry or plasma-based techniques is rather complicated. Thus, the CVD synthesis of diamond-based composite films with a second component different from SiC is of great interest to expand the class of materials for porous diamond preparation and add more flexibility in terms of variation in the film thickness, diamond quality and achievable pore sizes.

In our recent work,^62^ we discovered the co-deposition of polycrystalline germanium and diamond phases using microwave plasma CVD (MPCVD) in CH_4_/H_2_/GeH_4_ gas mixtures. Later we confirmed the possibility of using CVD synthesis for the controllable formation of diamond/germanium composites with high crystalline quality of both diamond and germanium phases, while also keeping the deposition rates as high as ∼1 μm h^−1^.^63^ Here, we report on the fabrication of porous microcrystalline diamond films by chemical etching of the Ge component in such diamond/germanium composites.

## Experimental

**Fig. 1:**
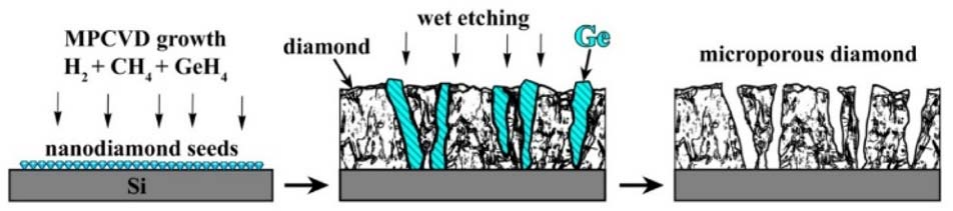
Scheme of porous polycrystalline diamond film fabrication.

The overall approach for the formation of porous diamond films from CVD-grown diamond–Ge composites is illustrated in Fig. 1. The diamond and germanium phases are co-deposited in a microwave plasma using CH_4_/H_2_/GeH_4_ gas mixtures. Then, the germanium phase is selectively removed by means of wet etching, which allows obtaining a diamond film with a three-dimensional network of micropore – porous diamond.

The substrates for different experiments on CVD growth of “diamond–Ge” composite films included (100)-oriented 10 × 10 × 1 mm^3^ polished Si substrates; CVD-grown polycrystalline diamond (PCD) films with a thickness of 50 μm, and a (100)-oriented 4 × 4 × 0.5 mm^3^ polished HPHT single-crystal diamond. To stimulate the formation of coalesced PCD films on Si substrates, they were seeded using a suspension of nanodiamond particles (particle sizes 3–7 nm, zeta potential > +50 mV, Cardiff University),^64,65^ by spin coating the wafers at 3000 rpm. This enhanced the diamond nucleation density to values in the order of 10^10^ cm^−2^.

The composite films were deposited in an MPCVD system ARDIS-100 (2.45 GHz, Optosystems Ltd, Russia)^66^ with CH_4_/H_2_/GeH_4_ gas mixtures. By default, the flow rates of the precursors CH_4_/H_2_/GeH_4_ were fixed at 20/478/2 sccm, so that the concentrations [GeH_4_] and [CH_4_] in gas were 0.4% and 4%, respectively. The pressure in the reactor was 65 Torr and input microwave power was 5.0 kW. However, in preliminary investigations, other concentrations [GeH_4_]/[CH_4_] (hereafter “Ge/C”) between 1% and 20% were also considered for comparison. The substrate temperature was kept at 800 °C as measured with a two-color pyrometer METIS M322 (SensorTherm GmbH).

For comparison reasons, 500 nm-thick diamond–SiC composite films were synthesized using similar growth conditions as for the diamond–Ge composite, but instead of germane, a monosilane (SiH_4_) gas was added into the gas mixture with concentration [SiH_4_]/[CH_4_] = 20%.^61,67^ Other CVD process parameters for growth of the diamond–SiC composite were: concentration of methane 4%, total gas flow 500 sccm, microwave power 5 kW, pressure 72 Torr, substrate temperature 800 °C, deposition time 2 hours, and growth rate ≈ 250 nm h^−1^. More details on such growth and the exact recipe are reported in ref. 61. The technique for the formation of diamond membranes was reported in ref. 68.

For a routine selective removal of the germanium phase, the as-grown diamond–Ge composite samples were treated in a mixture of concentrated acids HF : HNO_3_ (3 : 1, 99% pure, 60 °C, 1 min). For comparison purposes we measured the etching rate of single-crystal Ge, Si and 4H-SiC wafers under identical conditions as mentioned above. In case of Ge and Si a part of the sample area was protected by a Teflon (polytetrafluoroethylene) film mask, and after exposure to the etchant for 12 min and 0.7 min, respectively, the height of the step formed was measured with an optical profilometer (NewView 5000, ZYGO). Because of very slow etching of the SiC wafer, it was treated for 45 min without the mask and the etch rate was calculated from the weight loss.

The film surface morphology, grain size and cross section structure were examined using a Tescan MIRA3 scanning electron microscope (SEM), equipped with an energy-dispersive X-ray spectroscopy (EDX) module for elemental analysis. Raman and photoluminescence spectra were taken at room temperature with a LabRam HR840 (Horiba) spectrometer in a confocal configuration. The laser beam at 473 nm wavelength was focused in an ≈1 μm spot on the sample surface on different locations (Olympus BX41).

The thicknesses of the films were determined from the SEM images of their cross-sections. The calculated deposition rates of composite films were 1.1 ± 0.2 μm h^−1^, which is in good agreement with our previous study.^63^ Such high deposition rates allowed the formation of composite films with thicknesses ranging from <1 μm up to 200 μm.

## Results & discussion

### Formation of diamond–Ge composite films

**Fig. 2:**
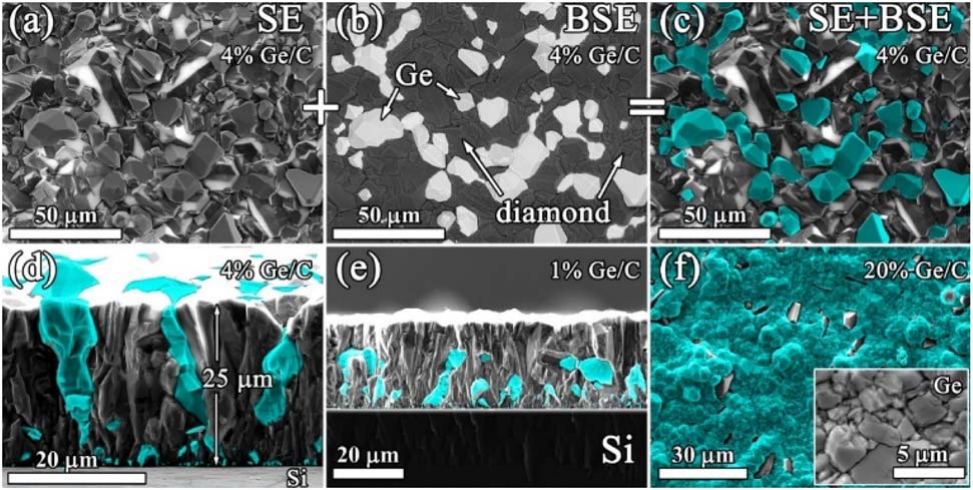
SEM images (plan views) of the polycrystalline diamond–Ge film taken in SE (a), BSE (b) and (c) combined SE + BSE modes; cross-sectional SEM images of diamond–Ge composites, grown at (d) Ge/C = 4% and (e) Ge/C = 1%. (f) Plan view SEM image of a diamond–Ge composite, grown at Ge/C = 20% (inset – structure of Ge crystallites with higher magnification). In images (c)–(f) the cyan color represents information about the location of Ge grains, obtained in the BSE mode. Note that images (a)–(c) show the same area of the sample.

The resulting structure of typical composite films of germanium with polycrystalline diamond (PCD–Ge) is demonstrated in Fig. 2. For the 25 μm-thick PCD–Ge film on a Si substrate, the secondary emission (SE) imaging mode of SEM reveals a polycrystalline structure with well faceted grains with a size of 10–20 μm (Fig. 2a). In backscattered electrons (BSE) mode, which enhances the contrast between different phases due to the high *Z*-contrast between carbon and germanium, the Ge crystallites are clearly recognized as light contrast areas (Fig. 1b, also see more details on the investigation of such PCD–Ge composites in ref. 63). Hereinafter, for convenience, the information on these two modes is combined in single pictures, in which the topography of the sample surfaces is taken from the SE images, while the information about the Ge content is based on BSE images and is shown in cyan color (compare Fig. 2a, b and c). The SEM images of the sample cross section reveal many submicron Ge grains on the film/substrate interface and more rare large Ge grains penetrated through the film thickness (Fig. 2d). However, changing the Ge/C content affects the proportion between PCD and Ge phases. As is known, the CVD growth of PCD films follows the van der Drift competitive model,^69^ in which the grains with a higher growth rate (which is caused by “fortunate” orientation) eventually grow over the tops of grains with lower growth rates. Here, we demonstrate, that in the case of co-deposition in CH_4_/H_2_/GeH_4_ gas mixtures, the diamond and Ge separate grains compete in the growth process as equals. Thus, managing the Ge/C ratio in the gas can be used to tune this competition, shifting the balance between diamond and Ge contents in the film. Lowering the Ge/C ratio down to 1% allows the majority of Ge grains to be eventually consumed by the PCD film (Fig. 2e). In contrast, raising the Ge/C up to 20% allows the formation of a continuous polycrystalline germanium film with just separate diamond grains visible (Fig. 2f).

**Fig. 3:**
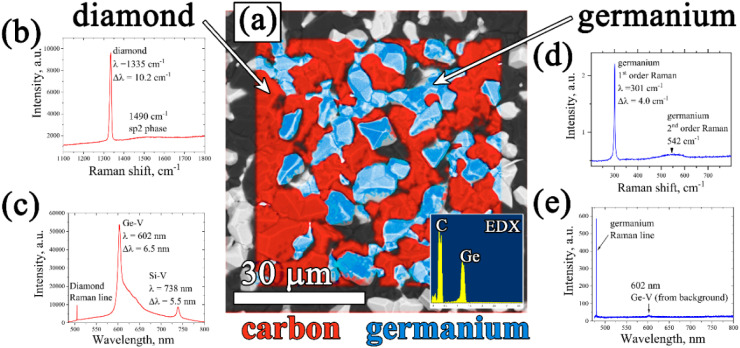
(a) EDX-mapping of the diamond–Ge film (inset – integrated EDX signal); Raman (b) and PL (c) spectra of the diamond component of the composite; Raman (d) and PL (e) spectra of the germanium component of the composite.

The EDX mapping of the surface of the composite film confirmed its two-phase structure, consisting of intermixed “all-carbon” and “all-germanium” grains (Fig. 3a).

The Raman spectrum (Fig. 3b) of the “all-carbon” part of the film reveals a narrow peak at 1334 cm^−1^, a fingerprint of diamond, shifted, however, from the normal position at 1332.5 cm^−1^ towards higher wavenumbers at 1334.0 cm^−1^ due to compressive stress in the film. The full width at half maximum (FWHM) is 4.7 cm^−1^. In addition, a wide band centered near 1515 cm^−1^ is present which is caused presumably by the superposition of the G peak at 1550 cm^−1^ (nondiamond sp^2^ carbon) and the 1480 cm^−1^ (*trans*-polyacetylene, *t*-PA) peak.^70^ Both the nondiamond carbon components are believed to be located mostly on grain boundaries.

The presence of GeH_*x*_ radicals in the CH_4_–H_2_–GeH_4_ plasma results in doping of diamond grains with germanium and formation of a germanium-vacancy defect that manifests itself in the form of a specific emission in the orange spectrum with a zero-phono line (ZPL) at 602 nm.^62^ The photoluminescence (PL) spectrum for the 25 μm film on Si is shown in Fig. 3c. Besides the diamond Raman peak at ∼504 nm, two other distinct features observed are the GeV band with a zero-phono line (ZPL) at 601.9 nm and the stronger SiV band with a ZPL at 738 nm. The SiV color center forms due to diamond doping with silicon as a result of Si substrate etching by atomic hydrogen of the plasma, and it is known to be quite a typical impurity in CVD diamond films.^71^

The Raman spectrum for a Ge phase was taken by focusing the laser beam on a randomly chosen Ge crystallite (Fig. 3d). It reveals a sharp (full width at half maximum – FWHM) and strong 1st order Raman peak of crystalline germanium at 301.0 cm^−1^ and a wide and weak band at around 550 cm^−1^ belonging to the 2nd order peak.^72^ The peak position at 301.0 cm^−1^ for the Ge grain was actually identical to that of the measured reference single crystal Ge wafer, and thus, the stress on the Ge grains on the growth surface was insignificant. It should be that due to high optical absorption of Ge at the laser wavelength,^73^ only the first few tens of nm close to the top of the film contribute to the Raman signal. The PL spectrum of the Ge phase (Fig. 3e) did not reveal any additional Ge-related peaks.

### Comparison of diamond–SiC and diamond–Ge composites

For this part of the study, MW CVD synthesis was used to grow, under similar conditions, diamond–Ge and diamond–SiC composites with similar (≈500 nm) thicknesses for the PCD part of composites. In both cases, the MW power was 5 kW, substrate temperature was 800 °C, and methane concentration was 4% (see more details in the Experimental section). However, growth rates varied significantly: 250 nm h^−1^ for the diamond–SiC composite and ≈1 μm h^−1^ for the diamond–Ge composite.

**Fig. 4:**
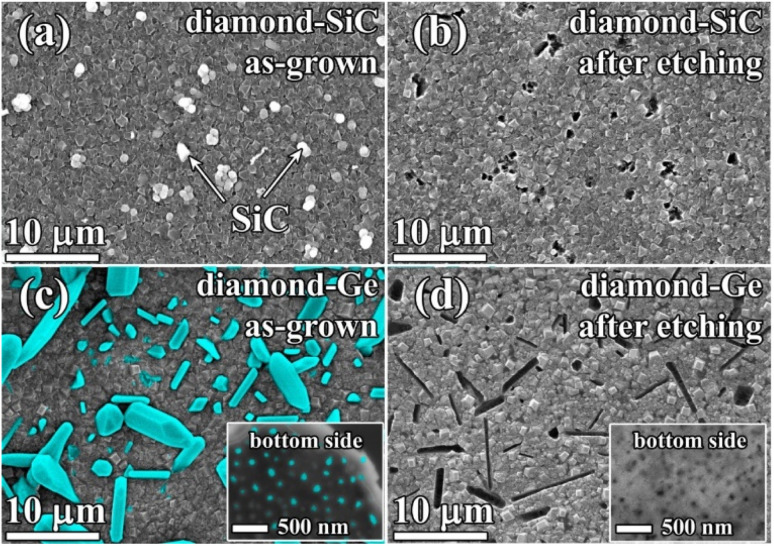
SEM images of 500 nm-thick films of the diamond–SiC composite before (a) and after (b) etching of the SiC component, and SEM images of 500 nm-thick films of the diamond–Ge composite before (c) and after (d) etching of the Ge component. Insets of (c) and (d): bottom (nucleation) sides of diamond–Ge membranes, taken before (c) and after (d) etching of Ge. Both (c) and (d) images were taken in “SE + BSE” mode; however, no signal from Ge was detected in (d).

The SEM image in Fig. 4 shows a plan view of diamond–SiC (Fig. 4a and b) and diamond–Ge (Fig. 4c and d) composite films before (Fig. 3a and c) and after (Fig. 4b and d) etching of non-diamond phases. In Fig. 4a, the white-contrast spheres are SiC crystallites immersed in a diamond matrix. The etching of SiC is a slow process, which took 24 h to ensure the complete removal of SiC even for such a thin composite film. To underline the etching time as a factor in favour of Ge-based diamond composites, we measured the etching rate of single-crystal Ge, Si and 4H-SiC wafers under identical conditions (Table 1). The measured etching rate for 4H-SiC is comparable with that reported for nanocrystalline 3C-SiC (3.3 nm min^−1^)^16^ and polycrystalline 3C-SiC thin films (0.01–5 nm min^−1^).^74^ In the case of the complex structure of diamond-based composites, the expected etching rates are even smaller as it takes more time for molecules of acids to reach the SiC/Ge phase which is buried deep in the bulk of the composite. Thus, removing SiC/Ge from thicker composite films will require much longer etching procedures, so the higher etching rates for Ge would be a great benefit. We note that for Si the etching rates are even higher, but CVD growth of the desired Si-diamond composite has not yet been reported by any group, so the values for Si are shown just for a comparison.

**Table 1:** The wet chemical etching rate in HF : HNO_3_ (3 : 1, at 60 °C) for Si, Ge and 4C-SiC separate crystals

Sample	Si	Ge	SiC
Etching rate, μm min^−1^	375 ± 5	11.6 ± 0.5	0.0032 ± 0.0005

In the diamond–SiC films after the full removal of the SiC phase (Fig. 4b), the regular array of pores with a size of ≈250 nm was observed at all areas of the sample surface. The density of such holes was 2–3 holes per 10 μm^2^. However, it is known from studies by other groups, that such a density of SiC grains can be controlled by varying CVD synthesis conditions and Si abundance in the gas.^16,75–77^

In the diamond–Ge composite (Fig. 4c), well-faceted large grains of crystalline Ge can be observed. To investigate the nucleation side of such a diamond–Ge composite, the Si substrate was selectively removed (see inset of Fig. 4c). The density of Ge sites, which were nucleated directly on the Si wafer, but not necessarily extended to the final growth surface, was ∼10^9^ cm^−2^.

After the chemical removal of Ge grains (Fig. 4d), the growth side demonstrated variously shaped holes with a size ranging from 100 nm to ≈10 μm (Fig. 4b). Still, the shape of the holes is in good correlation with the shape of Ge crystals seen in Fig. 4a. The nucleation side now displays porosity as a result of removal of the Ge particles grown directly on the Si substrate, with a pore size less than 100 nm (see inset of Fig. 4d). These images give examples of a variety of pore shapes and sizes achievable by growth and treatment of the Ge–diamond composites, which can also be affected by varying CVD growth conditions and the germane content in the gas.^62,63^

### Porous diamond membranes

**Fig. 5:**
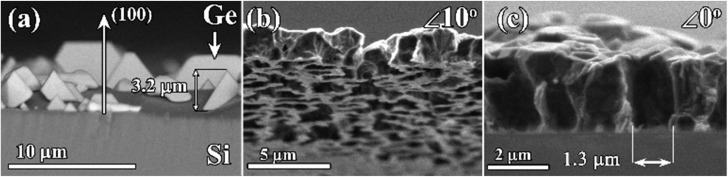
SEM images of (a) heteroepitaxial grains of Ge, grown on Si substrates using MW CVD in GeH_4_–H_2_ gas mixtures; (b) cross-section of the microporous PCD membrane (free standing) tilted by 10° towards the bottom side of the film; (c) non-tilted cross-sectional image of the free standing membrane with the demonstration of the pore size.

For some applications (*e.g.* high flow filtration), it is desirable to control the diameter of “end-to-end” pores through the diamond films or membranes. Thus, it is preferable to control the size of the initial Ge nuclei. To prevent Ge grains from being quickly overgrown by diamond grains (as in Fig. 2e), the growth of Ge grains on unseeded Si can be performed in H_2_–GeH_4_ gas mixtures without methane additions. The SEM image of the surface of the Si substrate after such a 2 h-long growth is shown in Fig. 5a. In this CVD process, heteroepitaxial Ge crystallites with sizes of few μm are formed. The (100) orientation of the observed Ge grains is similar to the (100) orientation of a monocrystalline Si substrate (*e.g.* compare with the work by Wang *et al.*^78^), thus confirming the heteroepitaxy of Ge (for additional XRD study of the PCD–Ge composite see ref. 63). No diamond grains were observed.

For the growth of composite films with a larger size of Ge grains, the Si substrates were initially seeded with nanodiamond powders and then subjected to two-step CVD growth conditions. The first stage of the CVD process was 5 min of Ge-only deposition in a GeH_4_–H_2_ gas mixture, after which the growth regime was changed to the standard regime in the GeH_4_–CH_4_–H_2_ mixture favorable to the deposition of the diamond–Ge composite to grow a 2.5 μm-thick composite diamond–Ge film. The SEM images of the resulting porous PCD membrane after removal of both the Si substrate and Ge grains are shown in Fig. 5b. The PCD film contains an irregular pattern of micrometer-sized holes. The SEM image of the cross-section of the membrane (Fig. 5c) suggests the formation of end-to-end holes (pores) with sizes of ∼1 μm.

### Porous films on diamond substrates

Another interesting process is the possibility of the formation of Ge grains and diamond–Ge composites not on Si or other substrates, but on diamond itself. For that, the investigated regimes for the formation of diamond–Ge composites were applied to polycrystalline and single crystal diamond substrates instead of seeded Si wafers.

**Fig. 6:**
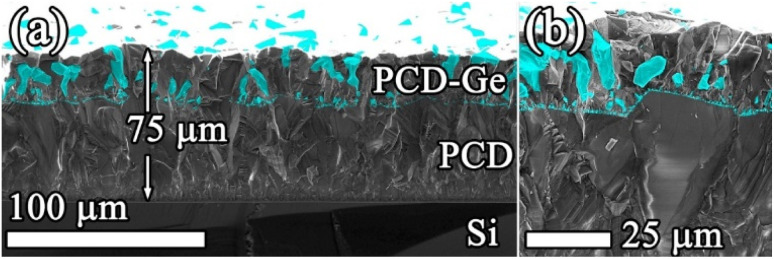
Combined “SE + BSE” SEM images of a cross-section of the diamond–Ge composite film grown on a PCD substrate: (a) general view; (b) detailed view of the film/substrate interface. Note that Ge crystallites directly nucleate on the large diamond grains.

A 25 μm thick composite film was deposited on a 50 μm thick PCD film that was preliminary grown on a Si substrate. The SEM image of the cross-section of the resulting sample is shown in Fig. 6a. Numerous fine Ge particles can be seen nucleating in the bulk of the PCD film, while their size steadily increases towards the surface of the sample. A closer look at the interface zone (Fig. 6b) shows large ∼20 μm faceted diamond crystallites on the substrate decorated with submicron nucleated Ge grains directed normally to the inclined diamond facets. The growth direction of the primary Ge grains with respect to the diamond facets seems to be independent of the facet orientation. The majority of the nucleated Ge grains appear to be buried inside faster growing diamond grains of the composite, yet some larger Ge grains continue to grow and eventually reach the composite top surface. Note that in Fig. 6b, the Ge grains split a large diamond grain into a few smaller ones, which means that such diamond–Ge sublayers may be used to influence the grain size and the roughness of thick PCD films (*e.g.* see ref. 79).

**Fig. 7:**
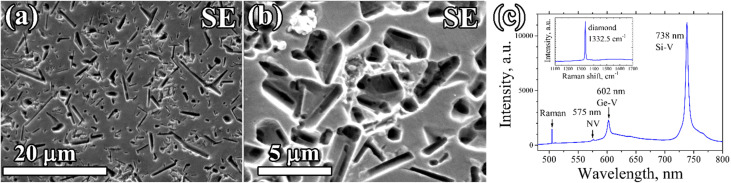
Porous epitaxial diamond film grown on a single-crystal HPHT diamond substrate: (a) and (b) SEM images at different magnifications. (c) PL spectrum of the CVD layer after the etching of Ge grains (inset – Raman spectrum).

A porous CVD diamond layer formed from the diamond–Ge composite film deposited on a polished (100) single crystal HPHT diamond is shown in Fig. 7. The diamond network in this case grew homoepitaxially to 2.5 μm retaining the smooth substrate surface relief, without any polycrystalline (non-epitaxial) diamond inclusions, thus indicating the single crystal structure of the porous layer. After etching, numerous rounded and rectangular holes formed, defined by shape of the Ge crystallites in the primary composite. The sizes and shapes of the holes were also similar to ones obtained for porous PCD films. Closer investigation of the formed pores shows that they have a complex micro-channelled structure (Fig. 7b).

The PL spectra of the porous single-crystal CVD layer (Fig. 7c) showed an intensive signal of germanium-vacancy color centers at 602 nm, which is another piece of evidence that the formed pores are formed in the epitaxial CVD layer, and are not a result of etching.

### Advantages of diamond–Ge composites

The three orders of magnitude higher etch rate for the Ge component illustrates the advantage of preparation of porous diamond from the diamond–Ge composites in comparison with the use of a diamond–SiC composite for the same purpose, the latter requiring a much longer time to remove the SiC grains.^16^ We note that the wet etching method we used favors the etching of the Si substrate after the full removal of Ge grains, which can influence the adhesion of the obtained films. Similarly, the selective removal of the Si substrate that was used to form diamond membranes leads to the removal of Ge grains as well. In those cases, a more selective yet slower wet/dry etching procedure can be used instead.^80–82^

The growth rates of diamond–Ge composites are also much higher than those of diamond–SiC films, which allows using diamond–Ge as a starting material for the formation of a thick (>100 μm) composite or porous material.

We confirmed this difference by growing both diamond–SiC and diamond–Ge composites in similar CVD regimes by changing only the 3rd gas – silane and germane, respectively. In the case of diamond–Ge deposition, the growth rate of the composite film increased by a factor of 4 with respect to that of the diamond–SiC composite. We attribute this difference to a higher abundance of added impurity gas for the diamond–SiC composite ([SiH_4_]/[CH_4_] = 20%) than for the diamond–Ge composite ([GeH_4_]/[CH_4_] = 4%), which could affect the gas chemistry in the plasma ball or the surface of the sample. Another factor is that the formation of the SiC phase requires both silicon and carbon atoms, which is not the case for the formation of germanium crystallites.

The size of Ge grains may be controlled by changing the GeH_4_/CH_4_ ratio in the gas (see Fig. 2) or by manipulation of the substrate temperature.^63^ Furthermore, both of these parameters might be varied during growth, similar to an approach for the diamond–SiC composite by Yuan *et al.*^83^ Another interesting option is based on the preliminary formation of Ge islands on the Si substrate before diamond deposition. Then, these pre-existing Ge grains continue to grow vertically after supply of methane in the plasma, being surrounded by competing diamond grains. In this case, the size of the primary Ge nuclei is controlled by the time delay of the CH_4_ supply. Such ordered Ge seeds could be grown on Si by selected-area deposition in open windows in a Si mask, as described by Li *et al.*^84^ Potentially, their method can be combined with our process for fabrication of an ordered diamond–Ge composite.

Finally, the formation of the diamond–Ge composite layer on the single-crystal diamond substrate allowed the formation of epitaxial porous CVD layers, which provides new perspectives in practical applications of porous diamonds. Specifically, they can be used as heat spreaders with enhanced cooling efficiency by means of liquid transport through the pores.^85^ Hydrogenated (treated in hydrogen plasma) porous diamonds demonstrate superhydrophobic surfaces.^25,26^ The undoped porous films are used for filtration, separation and extraction,^24^ while electrically conductive porous diamond is an excellent material for electrochemical electrodes.^52,86^ Although it is not expected that doping of diamond with Si or Ge atoms will essentially increase electrical conductivity of diamond, the co-doping of diamond with boron^87^ or phosphorus^88^ may be performed for that task.

## Conclusions

We developed a route to fabricate porous microcrystalline diamond films and homoepitaxial single-crystal diamond layers by synthesis of composite diamond–germanium films followed by chemical etching of the Ge component. The composite films were produced by co-deposition of diamond and crystalline Ge by MPCVD in CH_4_–H_2_–GeH_4_ mixtures on different substrates such as a Si wafer, PCD and single-crystal diamond plates, on which Ge grains spontaneously nucleate. The chemical etching of the Ge component is very fast, which provides a technological advantage to the porous diamond preparation process. Possible applications of the porous diamond films include thermal management with forced heat dissipation, surfaces with superhydrophobic properties, and chromatography.

## Author contributions

V. Sedov: conceptualization, funding acquisition, project administration, visualization, writing – original draft preparation, writing – review & editing. A. Martyanov: conceptualization, investigation, data curation, visualization, writing – review & editing. A. Popovich: investigation. S. Savin: investigation. D. Sovyk: investigation. I. Tiazhelov: investigation. D. Pasternak: resources. S. Mandal: resources, writing – review & editing. V. Ralchenko: validation, supervision, writing – original draft preparation, writing – review & editing.

## Conflicts of interest

There are no conflicts to declare.

## Supplementary Material
